# Successful Deep Inferior Epigastric Perforator Flap Harvest despite Preoperative Therapeutic Subcutaneous Heparin Administration into the Abdominal Pannus

**DOI:** 10.1155/2016/9168154

**Published:** 2016-08-29

**Authors:** Joseph W. Duncumb, Kana Miyagi, Parto Forouhi, Charles M. Malata

**Affiliations:** ^1^School of Clinical Medicine, University of Cambridge, Cambridge CB2 0SP, UK; ^2^Plastic & Reconstructive Surgery Department, Addenbrooke's University Hospital, Cambridge CB2 0QQ, UK; ^3^Cambridge Breast Unit, Addenbrooke's University Hospital, Cambridge CB2 0QQ, UK; ^4^Postgraduate Medical Institute, Faculty of Health Sciences, Anglia Ruskin University, Cambridge CB1 1PT, UK

## Abstract

Abdominal free flaps for microsurgical breast reconstruction are most commonly harvested based on the deep inferior epigastric vessels that supply skin and fat via perforators through the rectus muscle and sheath. Intact perforator anatomy and connections are vital for subsequent optimal flap perfusion and avoidance of necrosis, be it partial or total. The intraflap vessels are delicate and easily damaged and it is generally advised that patients should avoid heparin injection into the abdominal pannus preoperatively as this may compromise the vascular perforators through direct needle laceration, pressure from bruising, haematoma formation, or perforator thrombosis secondary to external compression. We report three cases of successful deep inferior epigastric perforator (DIEP) flap harvest despite patients injecting therapeutic doses of low molecular weight heparin into their abdomens for thrombosed central venous lines (*portacaths™*) used for administering primary chemotherapy in breast cancer.

## 1. Introduction

Deep inferior epigastric vessels provide the basis for the commonest abdominal free flaps used for breast reconstruction. Arising from the external iliac vessels, they supply the infraumbilical skin and fat via perforators through the rectus muscle and sheath. Perforator vascular anatomy of the DIEP flap has been well studied, with Saint-Cyr et al. [1–3] elucidating the spatial organisation of the perforating vessels and their delicate intraflap branches. Intact perforator anatomy and horizontal connections are vital for optimal flap perfusion [1–5]. Patients are therefore advised to avoid any preoperative interventions that may damage the perforators and their branches and thus compromise flap perfusion. The latter includes injecting prophylactic or therapeutic low molecular weight heparin (LMWH) into the anterior abdominal fat.

We have previously reported on the safety of performing abdominal free flap operations following neoadjuvant chemotherapy [6] even when this is complicated by central venous catheter thrombosis [7]. The incidence of thrombosis in implanted central venous catheters in oncology patients undergoing long-term chemotherapy is 6.4–7.3% [8, 9]. This is treated by anticoagulation, which may be delivered by instillation, flushing, or systemic administration [10, 11]. In some patients, however, line-related thrombosis propagates and leads to symptomatic occlusion of the subclavian or innominate veins and/or pulmonary embolization [7]. These patients are normally managed by therapeutic anticoagulation for at least 3 months, using daily subcutaneous injections of the LMWH, which are continued until the line is removed [12]. The most accessible injection site is the lower abdominal pannus, the very tissue needed for DIEP flap breast reconstruction. There has been no previous study on the effects of LWMH administration into the abdominal pannus on subsequent flap harvest and perfusion.

We report three cases of successful DIEP flap transfer for immediate breast reconstruction despite injections of therapeutic doses of enoxaparin into their abdominal fat 24–32 hours before microsurgical transfer. All three patients were treated by primary chemotherapy for breast cancer and were prescribed subcutaneous heparin for line-related thrombotic complications. They were referred for reconstructive consultations towards the end of their chemotherapy and had already been receiving subcutaneous injections into the abdominal fat when a decision regarding DIEP flap reconstruction was taken.

## 2. Case Reports

### 2.1. Patient 1

A 55-year-old female with multifocal grade III invasive carcinoma of the left breast had her right subclavian vein cannulated for primary chemotherapy. Six weeks later, she developed a left axillary vein thrombus and was commenced on 1.5 mg/kg (100 mg) enoxaparin once daily. This was self-administered subcutaneously into the lower abdomen. Following chemotherapy, she underwent a mastectomy and DIEP flap reconstruction. On the day prior to surgery, therapeutic enoxaparin was omitted having been last administered 32 hours before her operation. No bruising was visible immediately preoperatively. There were no bleeding problems intraoperatively and estimated blood loss was 800 mL. She was commenced on a prophylactic dose of enoxaparin (40 mg daily) for the duration of her admission starting 6 hours after surgery. She also required a 3-unit blood transfusion. Her total breast and abdominal drain output was 2.7 L of serosanguineous fluid during her 7-day hospital stay. There were no flap circulatory or bleeding problems at the donor or recipient sites.

### 2.2. Patient 2

**Figure 1:**
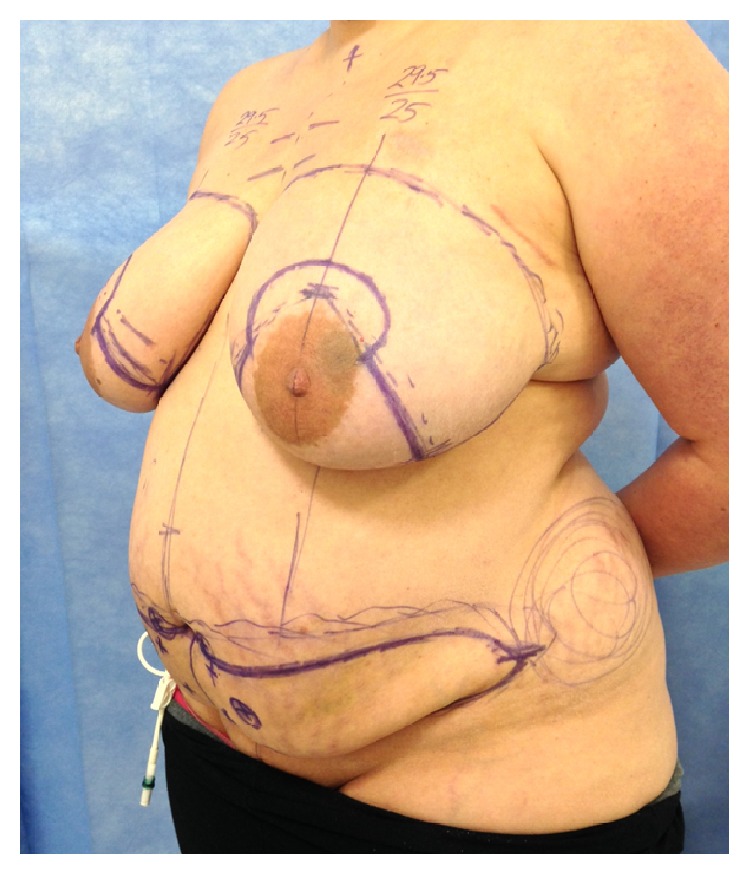
Preoperative photograph of a 33-year-old female (Patient 2) showing skin bruising on the left lower abdomen prior to bilateral mastectomy and DIEP flap reconstruction.

**Figure 2:**
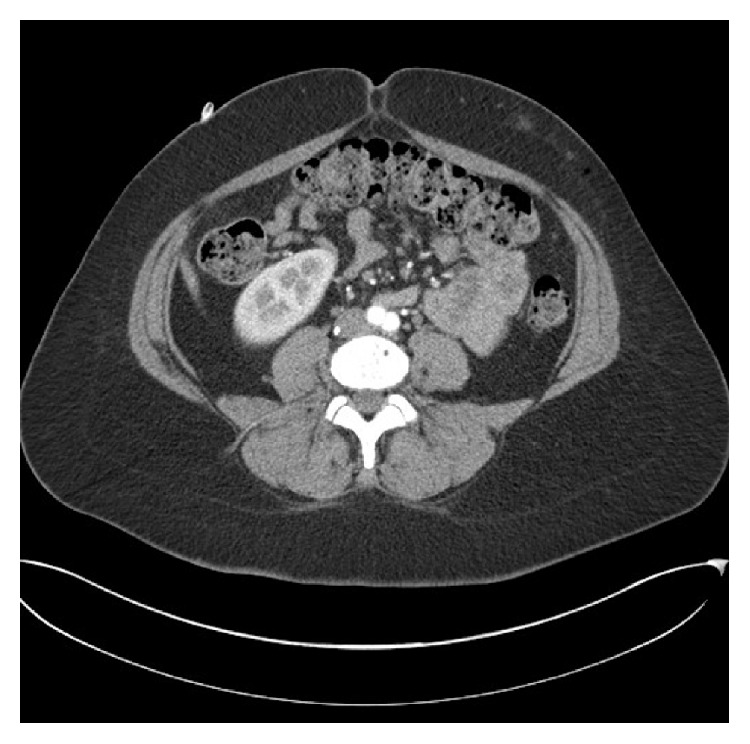
Preoperative CT angiogram of Patient 2 demonstrating subcutaneous opacities, representing subcutaneous bleeding from heparin injection over the left abdomen. Note the close proximity to DIEP perforators.

A 33-year-old female with grade III invasive ductal cancer was treated by primary chemotherapy to be followed by bilateral mastectomy (therapeutic on one side and prophylactic on the other side) along with bilateral DIEP flap reconstructions. Due to a history of port-related thromboses 13 years previously during treatment for ovarian cancer, she was put on prophylactic 40 mg daily subcutaneous enoxaparin. The heparin was injected into the lower abdomen above the groin creases and bilateral thighs whilst she had a femoral* Groshong* line for chemotherapy (Figure 1). A preoperative abdominal wall CT scan performed 48 hours before surgery (Figure 2) showed subcutaneous opacities consistent with bruising in the lower abdomen, particularly on the left side. The dose was reduced to 0.1 mg/kg (10 mg) in the two weeks prior to surgery and stopped the day before her surgery where an estimated 700 mL of blood was lost. Postoperatively, she received two units of blood and took 5,000 units of dalteparin daily for two weeks as thromboprophylaxis. Her drain output whilst in hospital is shown in Table 1. Both flaps were successful and there were no bleeding problems.

### 2.3. Patient 3

**Figure 3:**
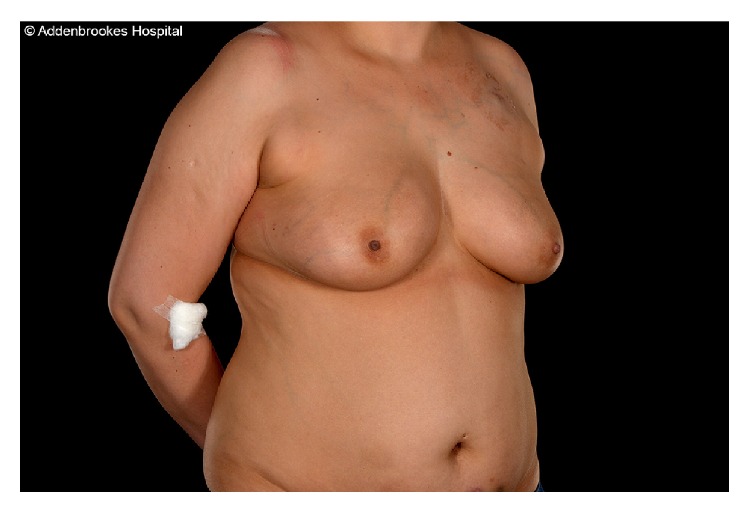
Preoperative photograph of a 37-year-old female (Patient 3) showing bilateral lower abdominal skin bruising at the sites of heparin injection prior to right mastectomy and DIEP flap reconstruction.

**Figure 4:**
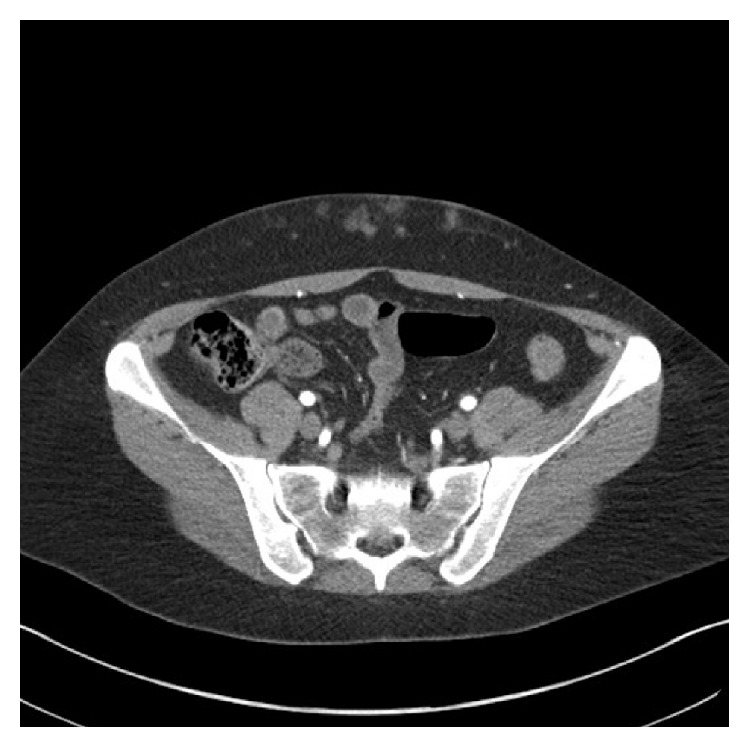
CT angiogram of Patient 3 showing multiple small subcutaneous haemorrhages bilaterally at the sites of heparin injections. DIEP branches appear intact in adjacent fat. The inferior epigastric arteries are seen running posterior to the rectus muscle and sheath.

A 37-year-old female with a grade II invasive ductal carcinoma developed a left subclavian port-related thrombosis one month after the start of 6 cycles of primary chemotherapy and was started on a course of therapeutic enoxaparin at 1 mg/kg (80 mg) daily, administered subcutaneously into her lower abdomen. Anticoagulation was stopped 32 hours before mastectomy and DIEP flap reconstruction. Preoperative photographs (Figure 3) demonstrated bruises in the lower abdomen that correlated with her CT scan findings 1 month prior to surgery of bilateral opacities consistent with soft tissue injury reported as representing bruising or inflammation of the subcutaneous fat (Figure 4). Intraoperative blood loss was estimated at 560 mL and she did not require a blood transfusion. Postoperatively, she was given 5,000 units of dalteparin subcutaneously daily for 6 days, before being discharged. Her flap remained healthy and healed well with no complications.

## 3. Discussion and Conclusion

Although there are currently no formal guidelines on the administration site of anticoagulants prior to free flap harvest, it is generally thought that administration of LMWH may damage the vasculature through local haemotoxic and mechanical effects. This could threaten flap viability, as quality and reliability of perfusion are partially dependent on intact subdermal plexus and direct or indirect linking vessel patency [1, 3, 4]^.^ It is equally important to try to avoid compromise to alternative nonintended perforators, just in case a rescue vessel is required.

We report three cases of successful abdominal free flap transfer for immediate breast reconstruction surgery after administration of subcutaneous heparin doses into the lower abdomen. In all cases, the last dose was administered approximately 32–36 hours prior to surgery, with none given on the evening before the operation. At prophylactic doses of subcutaneous heparin, there is no difference in bruising or APTT when using alternate injection sites on the thigh [13, 14] and upper arm [13] instead of the abdomen. However, the upper thigh should be avoided since the subfascial plane is a potential space which can allow haemorrhagic spread to the retroperitoneal space [15] and injections to the upper arm greatly inconvenience everyday activities. The anterior abdominal wall remains the site of choice for subcutaneous injections. We recommend that patients who may be potential candidates for DIEP flap reconstruction and their physicians should be educated on exact flap harvest location. Subcutaneous injections should be restricted to at least 5 cm above the umbilicus to avoid inadvertent damage to the flap perforators which are located in the infraumbilical abdominal pannus.

There has been a previous report of flap success despite accidental subcutaneous injection of low dose enoxaparin immediately adjacent to the perforators [16]. The authors commented that this was inappropriate and could have resulted in flap failure. Our cases extend the literature and confirm that local intraflap LMWH administration should not be considered an absolute contraindication for free flap harvest. If injections have been administered in the flap area, preoperative CT angiography [17] should be used to confirm the integrity of the intraflap vasculature prior to free tissue transfer. Despite clinically and radiologically evident bruising, while there is a theoretical risk of fat necrosis should perfusion be compromised, we have not experienced this in our clinical practice and estimate the probability of this to be low. To confirm the viability of the perforators selected for intraoperative use on CT angiography, we recommend the adjunctive use of a handheld Doppler probe immediately preoperatively. Importantly, it is also now our practice to advise patients to avoid injecting proximal to potential lower abdominal donor sites once a decision for DIEP flap harvest has been made and if local areas on one side appear bruised on clinical examination we consider using the vessels on the contralateral side, albeit keeping in mind the optimal perforator anatomy delineated by CT angiography.

## Figures and Tables

**Table 1:** Table of clinical data.

Patient	1	2	3
Age (years)	55	33	37
BMI (kg/m^2^)	25	40	29
Tumour Grade	III	III	II
Receptor status	ER+/HER2−	ER-/HER2−	ER+/HER2+
Reason for anticoagulation	Axillary vein thrombosis	History of port-related thrombosis	Subclavian vein thrombosis
Time to thrombus	6 weeks	NA	4 weeks
Enoxaparin dose (mg)	100	40	80
Duration of anticoagulation	3 months	2 months	4 months
Time LMWH stopped preoperatively (hours)	32 h	24 h (10 mg, 2 weeks earlier)	32 h
Operation side	Unilateral	Bilateral	Unilateral
Lymph node surgery	Level II ANC	Level II ANC	SLNBx
Flap weight (grams)	662 g	794 g (L), 805 g (R)	690 g
Perforators	Lateral row ×4	Medial row ×1 (L), lateral row ×2 (R)	Medial row ×1
Ischaemia time (mins)	97	79 (L), 80 (R)	26
EBL from swab weights (mL)	800	719	560
Thromboprophylaxis	40 mg enoxaparin	5000 units of dalteparin	5000 units of dalteparin
Total drain output (litres)	2.7 L	1.2 L	1.2 L
Hospital stay	7 days	7 days	6 days

EBL: estimated blood loss.

NA: not applicable.

ANC: axillary node clearance.

SLNBx: sentinel lymph node biopsy.

## Additional Points

This study was carried out at the Department of Plastic and Reconstructive Surgery, Addenbrooke's Hospital, Cambridge University Hospitals NHS Foundation Trust, Cambridge, UK.
